# Minding the Baby versus usual care: study protocol for a quasi-cluster-randomized controlled study in Denmark of an early interdisciplinary home-visiting intervention for families at increased risk for adversity

**DOI:** 10.1186/s13063-022-06434-2

**Published:** 2022-06-24

**Authors:** Maiken Pontoppidan, Mette Thorsager, Mette Friis-Hansen, Arietta Slade, Lois S. Sadler

**Affiliations:** 1 VIVE – The Danish Centre for Social Science Research, Herluf Trolles Gade 11, 1052 Copenhagen, Denmark; 2 Yale Child Study Center, 230 S Frontage Rd, New Haven, CT USA; 3 Yale University School of Nursing, 400 West Campus Drive, Orange, CT USA

**Keywords:** Pregnant, Infant, Mother, Interdisciplinary, Early intervention, Disadvantaged population, At risk family, Parent, Sensitivity, Parental reflective functioning, Mental health, Cluster-randomized controlled trial

## Abstract

**Background:**

Inequality in health can have profound effects on a child’s opportunities later in life. To prevent these downstream effects in families at increased risk of adversity, programs are needed to provide support and improve well-being across several domains. The present trial is aimed at assessing the effectiveness of the Minding the Baby® (MTB) home visiting intervention in improving the mother-child relationship, parental reflective functioning, well-being, and mental health, as well as child development and well-being in families at known risk of adverse health, relational, and developmental outcomes.

**Methods:**

The study is a pragmatic, prospective, quasi-cluster-randomized controlled trial in which seven Danish municipalities were randomized to MTB training in either 2018 or 2019. A total of 250 pregnant women at increased risk of adversity will be recruited (75 care as usual families and 175 intervention families). Care as usual families will be recruited before and after the MTB training. The MTB intervention is an attachment-based, interdisciplinary home visiting intervention offered from the third trimester of pregnancy until the child is 2 years old. The participants are assessed at baseline, and when the infant is 3, 12, and 24 months old. The primary outcome is maternal sensitivity measured by the Coding Interactive Behavior scale applied to video recordings of mother-infant interactions. Secondary outcomes include parent-child interaction, parental reflective functioning, parental mental health, maternal satisfaction, parental stress, and child development and well-being. The treatment effect is estimated as a fixed effect using a binary indicator of MTB treatment, and cluster-robust standard errors based on wild bootstrap are used for inference.

**Discussion:**

This is the first trial of MTB in a Scandinavian context and will include the largest sample yet in a trial of MTB. The trial is expected to contribute to knowledge about the effect of early support for pregnant women, their infants, and their families at increased risk of adversity.

**Trial registration:**

ClinicalTrials.gov NCT03495895. The study was registered on April 12, 2018.

## Administrative information

**Table:** 

**Title {1}**	Minding the Baby versus usual care: Study protocol for a quasi-cluster-randomized controlled study in Denmark of an early interdisciplinary home-visiting intervention for families at increased risk for adversity
**Trial registration {2a and 2b}.**	The study was registered at clinicaltrials.gov (NCT03495895) on April 12th, 2018.
**Protocol version {3}**	This is version 1.4 of the protocol
**Funding {4}**	The study is funded by the A.P. Møller Relief Foundation (den A. P. Møllerske Støttefond).
**Author details {5a}**	MP: VIVE –The Danish Center for Social Science Research, mpo@vive.dk MT: VIVE –The Danish Center for Social Science Research, meje@vive.dk MFH: VIVE –The Danish Center for Social Science Research, mfh@vive.dk AS: Yale Child Study Center LS: Yale University School of Nursing and Yale Child Study Center
**Name and contact information for the trial sponsor {5b}**	Maiken Pontoppidan, mpo@vive.dk, VIVE – the Danish Center for Social Science Research
**Role of sponsor {5c}**	The funding body did not help with the design, data collection, analysis, or interpretation of data and did not help in writing the manuscript or in the decision to submit the manuscript for publication.

## Introduction

### Background and rationale {6a}

Inequality in health can have profound effects on a child’s opportunities later in life [1, 2]. A recent register-based study including more than half a million Danish children aged 0–15 years found that children exposed to childhood adversity—such as material deprivation, loss within the family and negative family dynamics—have a markedly higher rate of hospitalization in childhood and adulthood [3]. The link between childhood adversity and later health problems is particularly strong for events such as injuries, unspecified symptoms, health service contacts, respiratory and infectious diseases, mental and behavioral diagnoses, and later pregnancy and childbirth [3] and highlights the need to support the well-being of families at increased risk of adversity to prevent health inequalities in the population.

Pregnancy and the transition to parenthood can be challenging for both parents and infants, particularly in families at increased risk of adversity. The fetal and infant brain is extremely plastic and infants need basic sensory, social, and emotional experiences and protection against toxic stress [4, 5]. Infants develop best in a responsive environment based on nurturing, consistent, and protective interactions with adults [6]. Fetal exposure to health risk factors during the mother’s pregnancy and exposure to abuse or neglect during the first years of life can cause long-term consequences such as poorer health, developmental problems, disrupted attachment, mental health problems, and poorer educational outcomes compared to the outcomes of children who are not exposed to those risk factors [7–11]. The quality of the early care that the infant receives is crucial, and the presence of a sensitive and responsive caregiver can protect the child from the negative influence of toxic stress [2, 12, 13].

Warm, sensitive, and responsive interactions between parents and their infants are crucial contributors to promoting a secure infant-parent attachment [14–16]. Results from studies across a range of countries and cultures suggest that approximately 40% of children have an insecure attachment to their primary caregiver [17]. Having an insecure attachment and, in particular, a disorganized attachment to the caregiver leads to a higher risk of later mental health and behavior problems [18–23]. The quality of the attachment bond depends on the sensitivity of the caregiver [14, 24–26]. Parental sensitivity refers to the parent’s ability to observe the child’s signals, to interpret them correctly, and to respond to them promptly and adequately [27]. This is key to the infant developing the capacities to regulate emotions and handle stress [15, 28], and as such, serves as a crucial protective factor as the child develops. Longitudinal studies show that positive, consistent, and supportive parenting predicts enhanced cognitive development and low levels of child problem behavior and child abuse [29–36]. If parents are supported in developing and applying sensitive parenting skills, healthy child development can be stimulated, maltreatment can be reduced, and future problems can be prevented [31, 33, 36–47]. Sensitive parenting also predicts lower costs to society later in the child’s life, irrespective of childhood level of poverty, antisocial behavior (on the part of the child), and IQ [48].

Recently, attachment-based parenting interventions have been developed to support the development of sensitive and secure attachment relationships between parents and infants. Studies find that parental sensitivity, parent-child interactions, and parent-child attachment can be improved through early intervention [37, 39, 49–56]. This is found especially in interventions that focus clearly on maternal sensitive behavior [57]. Often overlooked is the fact that the parent’s sensitive responsiveness depends upon their capacity to mentalize or reflect upon the child’s internal experience. The present study examines the intervention “Minding the Baby®,” which aims to both enhance parental sensitivity and to build the parent’s reflective capacities, namely their ability to understand their child’s intentions [58–60], to imagine or envision their child’s thoughts and feelings, and to understand their child’s behavior as a function of underlying subjective experience [58, 61].

Minding the Baby® (MTB) is an attachment-based, interdisciplinary home-visiting intervention aimed at improving developmental, health, and relationship outcomes in vulnerable young families having their first child. The MTB intervention aims to do so by (1) enhancing parents’ capacity to reflect on the child’s experience and thus respond sensitively, and (2) by providing the layers of emotional and concrete support necessary to support the parent-child relationship [62]. The intervention was first tested in two randomized controlled trials (RCTs) in the USA. In the first trial [63], which included 105 families, the intervention children—as compared to controls—were more likely to be securely attached and less likely to be disorganized in relation to attachment. In addition, child interactions at 4 months of age were less likely to be disrupted with teen mothers, and mothers at the highest risk improved in their reflective functioning over the course of the intervention. Participation in the intervention was also associated with several positive health and public health outcomes. The MTB families were more likely to be up-to-date with their pediatric immunizations at when the child turned 1 year, young mothers were less likely to experience rapid subsequent childbearing and children were less likely to be referred to Child Protective Services [63]. In a small follow-up study, MTB mothers reported less externalizing behavior at child ages 3–5 years [64].

In a second RCT [61], participation in MTB was linked to improved parental reflective functioning, higher levels of secure attachment, and lower levels of disorganized attachment when compared with controls. In a combined sample from the two studies, there were lower levels of obesity in toddlers from MTB families compared with control group families [65]. In a later follow-up study, children who had participated in MTB had lower levels of behavior problems, and MTB parents were more likely to parent in a supportive way [66].

A recent RCT of MTB in the UK including 148 young mothers found no effect of MTB on the primary outcome parental sensitivity but found that MTB led to a reduction in child behavior problems at age 2 [67]. They found no significant effects on attachment security, cognitive development, maternal mental health, and subsequent childbirths. In a secondary analysis, when mothers who were fully engaged in the intervention were compared with those who were not, the level of engagement was linked to attachment security. Likewise, a secondary analysis revealed that while there were no main effects on parenting stress, there was a treatment × time interaction, such that intervention mothers experienced less parenting stress between their child’s first and second birthday.

The mixed results from the two studies warrant larger intervention studies. The MTB intervention was originally developed for young mothers; both the US study and UK studies targeted mothers between the ages of 14 and 25. Becoming a teenage mother is a rare event in Denmark (0.45% of all births in 2020) and even becoming a mother before age 25 is relatively rare (8.15% of all births in 2020) [68]. The target group for MTB in Denmark is, therefore, older than in the previous studies. Furthermore, MTB has not previously been implemented and tested in a Scandinavian welfare context. Consequently, it is important to examine the effects of MTB in a Danish context with an older population of pregnant women and mothers who are at risk for adversity.

### Objectives {7}

The trial aims to assess the effectiveness of the Minding the Baby® home-visiting intervention in improving parental sensitivity, parent-child interaction, parental reflective functioning, parental mental health, maternal satisfaction, parental stress, and child development and well-being. The hypothesis for the primary outcome is that mothers in the intervention group will have a higher level of parental sensitivity than mothers in the control group when the child is 12 and 24 months old. For the secondary outcomes, we hypothesize that mothers in the intervention group will show improved parent-child interaction, improved levels of reflective functioning, fewer signs and symptoms of mental health disturbance, higher maternal satisfaction, and lower parental stress, and that we will see improved child development and well-being.

### Trial design {8}

The study is a pragmatic, prospective, parallel, superiority, quasi-cluster RCT with two study arms: intervention (MTB) and care as usual (CAU).

## Methods: participants, interventions, and outcomes

### Study setting {9}

The study is conducted in 11 Danish municipalities. Most of the municipalities are medium-sized with populations ranging from 45,600 to 89,800, but the trial also includes three small municipalities with populations ranging from 26,000 to 38,000, and one larger municipality with a population of 350,000. Seven municipalities were randomized in 2018 on a site level to either the first wave (2018) or the second wave (2019) of clinical training and implementation of the intervention. Because recruitment was slower than expected, we added four municipalities to the study in 2019 and 2020. These four municipalities could not be randomized because there was only one clinical training session available when they joined the study. All municipalities recruit CAU participants from the beginning of the recruitment period and until they receive training. Once the clinicians have received training in MTB, they start recruiting intervention families. To secure enough CAU families, most municipalities continue to recruit CAU participants after receiving MTB training. To avoid contamination, municipalities have been asked to not let MTB clinicians handle control families.

Table 1 shows which year the 11 municipalities received clinical training and whether they were randomized or not.

**Table 1:** When the participating municipalities received training and whether they were randomized or not

Municipality	Randomized	MTB Teams	Training in 2018	Training in 2019	Training in 2020	Population
A	Yes	1	x			Small
B	Yes	2		x		Medium
C	Yes	2		x		Medium
D	Yes	2	x			Medium
E	Yes	2		x		Medium
F	Yes	0		(x)		Small
G	Yes	4	x	x		Big
H	No	1				Medium
I	No	2		x		Medium
J	No	2			x	Medium
K	No	2			x	Small

(x) the municipality withdrew from the study before training in 2019. Small: population <40,000; medium: population ≥40,000 and <100,000; big: >250.000

**Fig. 1:**
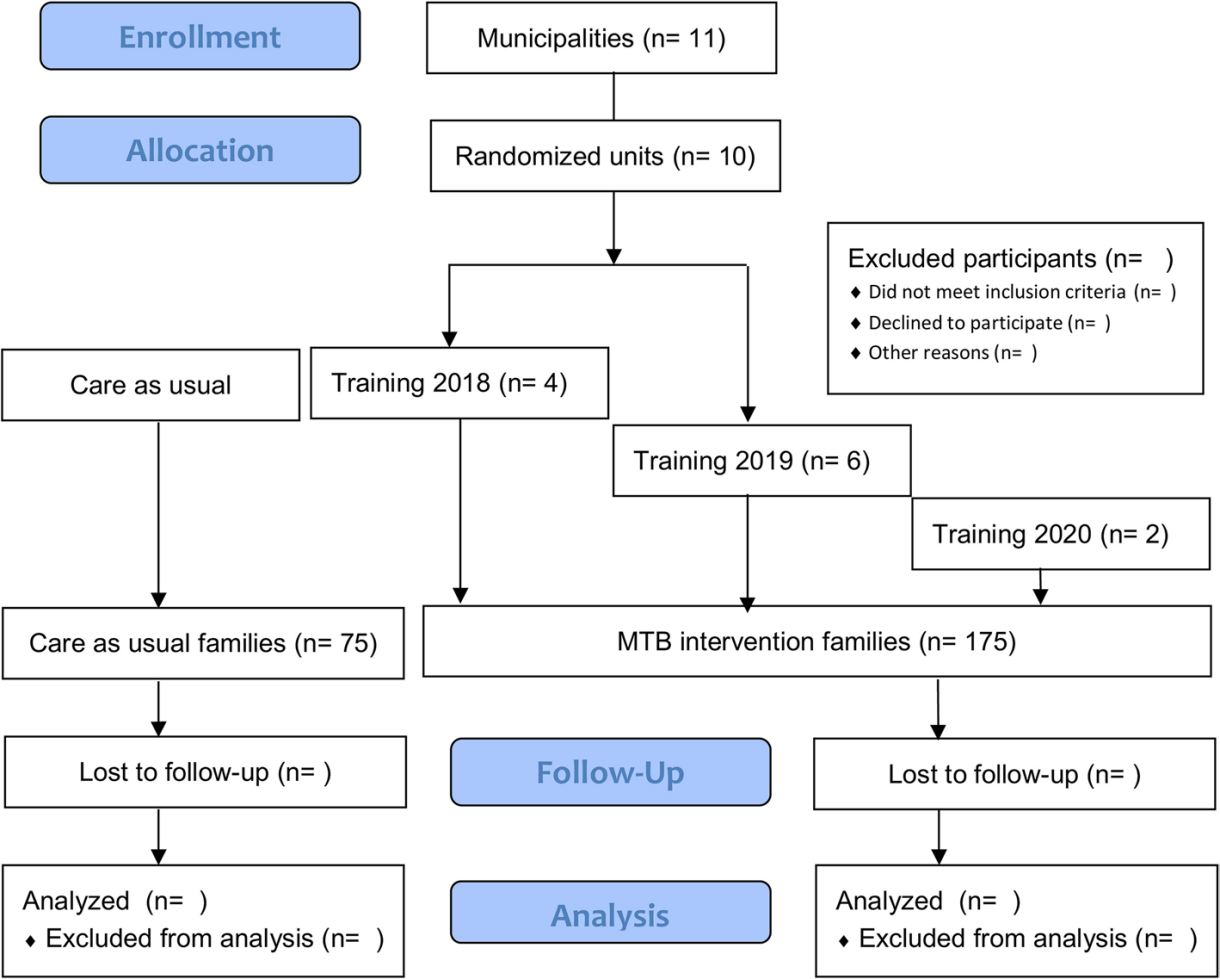
Study flow chart

The flow chart is presented in Fig. 1.

### Eligibility criteria {10}

#### Inclusion and exclusion criteria

Pregnant women at least 15 years old who are characterized as antenatal care groups 3 or 4 according to the Danish Health Authority’s recommendations for antenatal care [69] are invited to participate in the study. Pregnant women I care groups 3 and 4 have complex physical, mental, or social problems, or harmful substance abuse and need intensive care from an interdisciplinary team as they have a higher risk of preterm birth or pregnancy complications [70–72]. The pregnant women in the study may experience one or more the following characteristics: the authorities worry about the well-being of the child, the family has no support network, there is a history of childhood trauma or neglect, parental depression or anxiety, social problems, economic problems, unstable family relationships, and alcohol or substance abuse. Participation in the MTB intervention is voluntary.

Women are excluded if they fulfill one or more of the following criteria: (1) a child older than 3 years old living full-time with the family, (2) current severe substance abuse, (3) severe psychotic illness (F20), (4) profound or severe learning disabilities, (5) life-threatening illness in parent or child, and (6) not able to fill out questionnaires in Danish.

### Who will take informed consent? {26a}

Informed consent for participation in the study is obtained by municipal frontline workers after informing the pregnant woman about the study and receiving oral consent.

### Additional consent provisions for collection and use of participant data and biological specimens {26b}

Not applicable as this trial does not involve collecting biological specimens for storage.

### Interventions

#### Explanation for the choice of comparators {6b}

The comparison group receives the usual care that was being provided to families in the target group before MTB was implemented. According to Danish law, municipalities must offer support to families within this target group. Therefore, it is not possible to include a non-treated control group. As the target group for MTB is relatively small in many of the municipalities, they do not necessarily have other specific interventions to offer. We expect that CAU will differ between the participating municipalities.

#### Intervention description {11a}

##### Care as usual

Families in the control group receive the usual care that is offered to families in the target group in each of the participating municipalities. Universal prenatal care consists of four to seven midwife consultations, three general practitioner consultations, and two ultrasound scans [73]. At-risk pregnancies receive additional care based on individual needs including consultations with a social worker, medical doctor, and/or a therapist at a family clinic. The majority of uncomplicated births in Denmark are midwife-assisted hospital births. After hospital discharge, the municipality of residence is informed about the birth and the family gains access to home visiting by municipal health visitors. Home visits are provided by the municipality within the guidelines issued by the Danish Health Authority [74]. The standard package offered to all families includes one home visit during pregnancy and three to five visits during the first year of life. All Danish health visitors are registered nurses with 1.5 years of additional specialized training in supporting maternal, child, and family health.

All families are also offered a birth checkup and three well-child checkups with their general practitioner within the first year of the child’s life and when the child turns 2. The support offered to families in need of extra care consists of extra visits from health visitors, sessions with family therapists, and/or group or individual-based interventions such as the Circle of Security – Parenting (COS-P). Family therapists can include licensed psychologists or social workers and teachers with therapeutic training. Compared with MTB, usual care will often be commenced later (often after the child is born) and can be of higher intensity, but this will usually be for a shorter period compared to MTB.

We will register the type and intensity of interventions offered to the CAU group between the participating municipalities.

##### Minding the Baby

Based on an applied research model grounded in attachment and social ecology theory, Minding the Baby® (MTB) involves an integrated model of care that bridges primary care and mental health approaches to enhancing the mother-infant relationship and, whenever possible, the father-infant relationship [75]. The MTB program is aimed at mothers, children, and families at particular risk of impaired parent-child, health, life course, and mental health outcomes. MTB is focused on (1) enhancement of the parent-child relationship, the health, and mental health of mother and child, and life course outcomes within young families; and (2) prevention at a very early stage of the family’s development. Home visiting is the primary intervention modality, and visits begin in pregnancy through the child’s second birthday. All clinicians are trained in supporting and enhancing parental reflective functioning (PRF).

The core of the model consists of three key elements: (1) promoting secure attachment, parental reflectiveness, health and mental health, self-efficacy in the parent and infant; (2) Supporting parental reflectiveness through relationships with home visitors; and (3) using an interdisciplinary approach in meeting young families’ diverse and multiple needs.

Families are visited weekly beginning in the mother’s second or third trimester of pregnancy up through the child’s first birthday, and on a biweekly basis from the child’s first birthday through the child’s second birthday. Visits are carried out on an alternating basis by a team comprising a nurse (who is a trained health visitor) and a family therapist. Home visits last approximately 1 h, although this can vary based on a family’s particular needs. At times of crisis or when families require supplies (e.g., children’s clothes) or time, home visits can be extended or increased in frequency.

The MTB approach has been manualized around a well-developed set of principles, protocols, and guidelines contained within a treatment manual (available in English and Danish) (Slade et al., 2018). Clinicians must participate in a 4-day training course and, over the first 3 years of implementation, participate in monthly supervisory sessions with local supervisors and program consultants, in addition to ongoing educational sessions and fidelity oversight from program developers. Within the intervention design, the clinical work with individual families is flexible, individualized, and shaped by the family’s needs and circumstances at the time of each home visit. The clinicians confer regularly about each of their shared families; they also maintain close contact with health providers at the community health clinics from which families are recruited. All Danish MTB home visitors carry a part-time MTB caseload consisting of two to six families, except for one municipality where home visitors are full-time with MTB and carry a caseload of 22–25 families.

All intervention families will receive MTB in addition to care as usual. To increase consistency for the families, the MTB health visitors, in addition to their MTB work with caseload families, will conduct the routine visits prescribed by Danish health service mandates during the infant’s first 2 years.

#### Criteria for discontinuing or modifying allocated interventions {11b}

Women are withdrawn from the study if the child is placed in out-of-home care and the event will be registered as an outcome. If the participant does not wish to continue or decides to move to a municipality not offering MTB, the intervention will be discontinued. The intervention will also be discontinued if the MTB team cannot establish contact with the participant despite multiple attempts to do so over an extended period.

#### Strategies to improve adherence to interventions {11c}

Clinicians follow the treatment manual (available in English and Danish) in a flexible and individualized way that is shaped by the family’s needs and circumstances. Adherence to the manualized intervention will be ensured by regular supervision that includes regular home visitors’ team meetings, local supervision for case discussion, and reflective supervision, as well as consultation from trainers and model developers for case presentations, clinical discussions, review of difficult clinical scenarios, implementation of essential elements of the model, and ongoing education about topics relevant to the model application. After each visit, the MTB clinician will complete a short questionnaire about visit characteristics such as length, location, family members present, and the type of information and support they have offered to the family.

#### Relevant concomitant care permitted or prohibited during the trial {11d}

Participants are allowed to receive any other care during the trial. However, municipalities are asked not to offer additional therapeutic parent interventions (such as the Circle of Security) while parents are in MTB.

#### Provisions for post-trial care {30}

N/A—we will not offer any post-trial care.

### Outcomes {12}

Data are collected at five time points: T0: baseline immediately after recruitment, T1: baseline part two before the child is born, T2: when the infant is 3 months old, T3: when the infant is 12 months old, and T4: when the infant is 24 months old.

#### Measures/outcomes

Table 2 shows the timing of the measures.

**Table 2:** Socio-demographic measures assessed at T0–T4 include the mother’s age, education, occupation, ethnicity, number of children, household status, and housing situation

		T0	T1	T2	T3	T4
Parent measures						
Socio-demographic measures	Age, education, etc.	√				
Pregnancy reflective functioning	PRFQ-P	√				
Hospital Anxiety Depression Scale	HADS	√				
Experiences in close relationships	ECR-R	√				
Childhood trauma experience	CTQ		√			
PTSD symptoms	PTSD-8		√			
Well-being	WEMWBS	√		√	√	√
Well-being	WHO-5					√
Postnatal depression	EPDS			√	√	√
Maternal satisfaction and experience	BaM-13			√		
Parental reflective functioning	PRFQ				√	√
Parental Stress	PSS				√	√
Partner relationship	CSI4					√
Parenting	SEAM family profile					√
Overall health and life satisfaction		√		√	√	√
Loneliness and network		√		√	√	√
Breastfeeding expectations and duration		√		√	√	
Use of alcohol, drugs, and medicine		√		√	√	√
Household economy		√		√	√	√
Mobile phone					√	√
Birth control					√	
Experience with cross-sectional collaboration					√	√
Job expectations					√	√
Child measures						
Development	ASQ-3			√		
Social-emotional development	ASQ-SE2			√	√	√
Social-emotional development	SDQ					√
Language and communication	2-5					√
Child temperament				√	√	
Child health		√		√	√	√
Screen time child						√
Child care					√	√
Parent-child relationship measures						
Mother and baby interaction	MABISC				√	
Mother and infant interaction	MIRS					√
Learning activities	**S**inging, reading				√	√
Parent-child interaction (video)	CIB				√	√

#### Baseline measures

In addition to the socio-demographic measures, we include the following measures at baseline to assess the initial level and to account for them as possible moderators or confounders in the effect analyses.

The Prenatal Parental Reflective Functioning Questionnaire (P-PRFQ) [76] is a 14-item measure of *parental reflective functioning* or the pregnant woman’s ability to mentalize. The P-PRFQ is an adaptation of the PRFQ [77] and consists of three subscales: opacity of mental states (4 items), reflecting on the fetus-baby (5 items), and the dynamic nature of mental states (5 items). Cronbach’s alpha is 0.77 for the total score and 0.69–0.77 for the three subscales. Responses are on a Likert scale ranging from 1 to 7 with three different scales: (1) High-Low where 7 = optimal PRF, 1 = low PRF; (2) Low-High where 1 = optimal PRF, 7 = low PRF, and (3) Middle where 4 = optimal PRF, 1 and 7 = low PRF. The total score range is 7–98.

The Hospital Anxiety and Depression Scale (HADS) [78, 79] is a 14-item measure of *anxiety and depression*. HADS consists of the two subscales “anxiety” and “depression,” both ranging from 0 to 21, where low scores indicate less anxiety and depression.

The Experiences in Close Relationship Scale-Short Form (ECR-S) [80] is a 12-item measure of *adult attachment* consisting of the two subscales: “Anxiety” (fear of abandonment and a craving for interpersonal closeness) and “avoidance” (fear of intimacy and interpersonal dependency). Each subscale ranges from 1 to 42, where low scores indicate better attachment.

The Childhood Trauma Questionnaire (CTQ) [81] is a 28-item measure of adverse childhood experiences (ACE). Items are rated on a 5-point Likert scale (Never true, Rarely true, Sometimes true, Often true, Very often true). The CTQ consists of five subscales: emotional abuse, physical abuse, sexual abuse, emotional neglect, physical neglect, and a minimization/denial subscale. Each subscale ranges from 5 to 25, where low scores indicate less trauma experience.

The PTSD-8 inventory [82] is an 8-item measure of *posttraumatic stress disorder (PTSD) symptoms*, including intrusion, avoidance, and hypervigilance. The total score range is 8–32, where a low score indicates a lower level of PTSD.

#### Primary outcome

The primary outcome is *maternal sensitivity* measured by the Coding Interactive Behavior (CIB) instrument [83] when children are 12 and 24 months of age. The main hypothesis is that mothers in the intervention group will have a higher level of parental sensitivity (a CIB composite) than mothers in the control group. Maternal sensitivity is a subscale of the CIB. The CIB is a global rating system for social interactions that includes 22 parent codes, 16 child codes, and five dyadic codes rated on a scale of 1 to 5 which can be aggregated into the following composites: sensitivity, intrusiveness, limit setting, involvement, withdrawal, compliance, dyadic reciprocity, and dyadic negative states. The CIB is coded based on a 6-min mother-infant free play interaction recorded in the home or at another location preferred by the family. Adverse events will be monitored during intervention and reported to the PI. The PI will report severe and serious adverse events to the internal review board. The CIB system has been validated as an assessment measure in multiple studies of mother-child interactions in both normative and high-risk populations and shows stability over time, predictive validity, and adequate psychometric properties [50, 51, 83–85]. Mother-infant interactions are coded by reliable coders blind to treatment allocation. The inter-coder agreement will be calculated on a 10% randomly selected subset of the sample.

#### Secondary outcomes

*The parent-child relationship* will be measured by the remaining composites of the CIB: intrusiveness, limit setting, involvement, withdrawal, reciprocity, and negative states.

The short version of the Warwick-Edinburgh Mental Well-being Scale (WEMWBS) [86, 87] is a seven-item measure of *maternal mental health*. A total score is calculated by summing the seven items and converting the raw score according to a published conversion table. Raw score and converted score range from 7 to 35. Higher scores indicate better maternal mental health.

The World Health Organization (WHO)-5 Well-Being Index [88, 89] is a five-item measure of current *mental well-being*. Items that are rated on a six-point Likert scale (all the time, most of the time, slightly more than half the time, slightly less than half the time, some of the time, at no time). The total score ranges from 0 to 100, where a high score indicates better well-being.

The Edinburgh Postnatal Depression Scale (EPDS) [90, 91] is a ten-item measure of mothers’ *depression symptoms*. Depression total score ranges from 0 to 30. A low score indicates fewer depression symptoms, and a score of 11 or above is considered clinically significant.

Being a Mother (BAM-13) [92] is a 13-item measure of a *woman’s satisfaction and experience with being a mother*. Items are rated on a four-point scale (no, hardly ever; no, not very often; yes, some of the time; yes, most of the time). The total score ranges from 0 to 39. A low score indicates higher satisfaction.

The Parental Reflective Functioning Questionnaire (PRFQ) [77] is an 18-item measure of *parental reflective function*. The PRFQ consists of three subscales with score ranges from 6 to 42: (1) Pre-Mentalizing Modes (PRFQ-PM) with six items (a low score indicates better RF); (2) Certainty about Mental States (PRFQ-CMS) with six items (a midrange score indicates better RF); and (3) Interest and curiosity in mental states PRFQ-IC with six items (a high score indicates better RF).

The Parenting Stress Scale (PSS) [93, 94] is an 18-item measure of *parenting stress* that is rated on a five-point scale (strongly disagree, disagree, undecided, agree, strongly agree). The PSS consist of two subscales: Parental Stress (items 3, 4, 9, and 10–16) and Lack of Parental Satisfaction (items 1, 2, 5, 6, 7, 8, 17, and 18). When scoring the subscales LPS items are reversed, and item responses are dichotomized into 0 (strongly disagree and disagree) and 1 (undecided, agree, and strongly agree) and items 2 and 11 are left out. Scores are then summed to subscale scores each ranging from 0 to 9 (PS) and 0 to 7 (LPS), where a low score indicates less stress and higher satisfaction.

The Couple Satisfaction Index [95] is a four-item measure of relationship satisfaction. Items are rated on a 5-point Likert scale (not at all true to completely true). Total scores range from 5 to 20, with higher scores indicating higher relationship satisfaction.

The SEAM Family Profile [96] is a 17-item measure of *family characteristics* such as parental worries and need for additional support. Items are rated on a 3-point scale (most of the time; sometimes; do not know or do not know yet).

The Ages and Stages Questionnaire 3 (ASQ:3) [97] is a 30-item measure of *child developmental progress*. The ASQ:3 consists of the following five subscales: communication, gross motor, fine motor, problem-solving, and personal-social. Cronbach’s alpha ranges from 0.67 to 0.85 for the five subscales for the version for children aged 3 months [98]. The total score range is 0–300 and low scores indicate better development.

The Ages and Stages Questionnaire-Social Emotional 2 (ASQ:SE-2) [99] is a measure of *child social-emotional development*. The ASQ:SE-2 consists of the following seven subscales: self-regulation, compliance, social-communication, adaptive functioning, autonomy, affect, and interaction with people. Total score range is 0–150 (3 months 15 items), 0–260 (12 months 26 items). A low score indicates better development. Cronbach’s alpha ranges from 0.71 to 0.91. Concurrent validity ranges from 71 to 90%. Sensitivity ranges from 78 to 84%, and specificity ranges from 76 to 98% [100].

The Strengths and Difficulties Questionnaire (SDQ) [101, 102] for parents of 2-to-4-year-old children is a 25-item measure of *child behavior and psychopathology*. Items are rated by parents on a 3-point scale (not true, somewhat true, certainly true). The SDQ consists of five domains: hyperactivity/inattention, peer problems, conduct problems, emotional symptoms, and pro-social behaviors. The SDQ also has an additional seven-item impact supplement about daily function.

The 2–5 questionnaire [103] focuses on the development of 2-to-5-year-old children. The full questionnaire consists of 124 items. We include the seven-item language comprehension subscale and the ten-item spoken language subscale. Items are rated on a 3-point scale (does not apply; applies sometimes or to some extent; applies). The total score range for language comprehension is 7–21 and 10–30 for spoken language. A lower score indicates better language skills.

The Mother and Baby Interaction Scale (MABISC) [104] is a ten-item measure of *the mother-infant relationship* that is rated on a 5-point Likert scale (always, most of the time, occasionally, not often, never). The total score range is 0–40, and a high score indicates a better relationship.

The Mother-Infant Relationship Scale (MIRS) [105] is a 19-item measure of *distorted maternal representations*. Distorted maternal representations are disturbed thoughts and feelings that a mother may have about her infant and herself as a parent that influence parenting behaviors and caregiving. Items are rated on a 4-point Likert scale (never to always). The total score range is 15–45, where a low score indicates less distorted representations.

Activities with child consist of 4 items measuring *parent and child interaction* through activities such as singing and reading. The total score range is 4–24. A high score indicates more interaction.

The mothers will also be asked single items about overall health, life satisfaction, breastfeeding intention, breastfeeding duration, use of birth control, supportive network, loneliness, and how they experience the interdisciplinary collaboration between all clinicians involved (e.g., midwife, general practitioner, health visitor, family therapist). If the mother is not employed, she will be asked questions about indicators for progression toward employment (belief in own skills, mastery of health, and work identity). These items were developed in the employment indicator project [106].

#### Administrative data

When all children have turned 2 years old, we will retrieve data on all families from Danish administrative data such as the Population Statistics Register, the National Patient Register, the National Prescription Register, the National Health Insurance Service Registry, Oral Health Register, the Education Register, and the Danish Rational Economic Agents Model (DREAM). With these data, we can examine outcomes such as vaccinations, child examinations at the general practitioner and dentist, use of social and health care services including emergency visits, use of medication, and information about housing, education, and labor market participation for the parents.

#### Fidelity

In addition to the elements of interventionist training and ongoing supervision for fidelity monitoring, implementation process variables will be collected from each site. For intervention families, the MTB clinician must register each visit with the family. For all families, we will collect data from the municipality on what they have been offered (apart from MTB). We ask for information about who is providing the support and the intensity of the support (number and length of sessions). We also ask if the child has been placed in out-of-home care.

### Participant timeline {13}

**Fig. 2:**
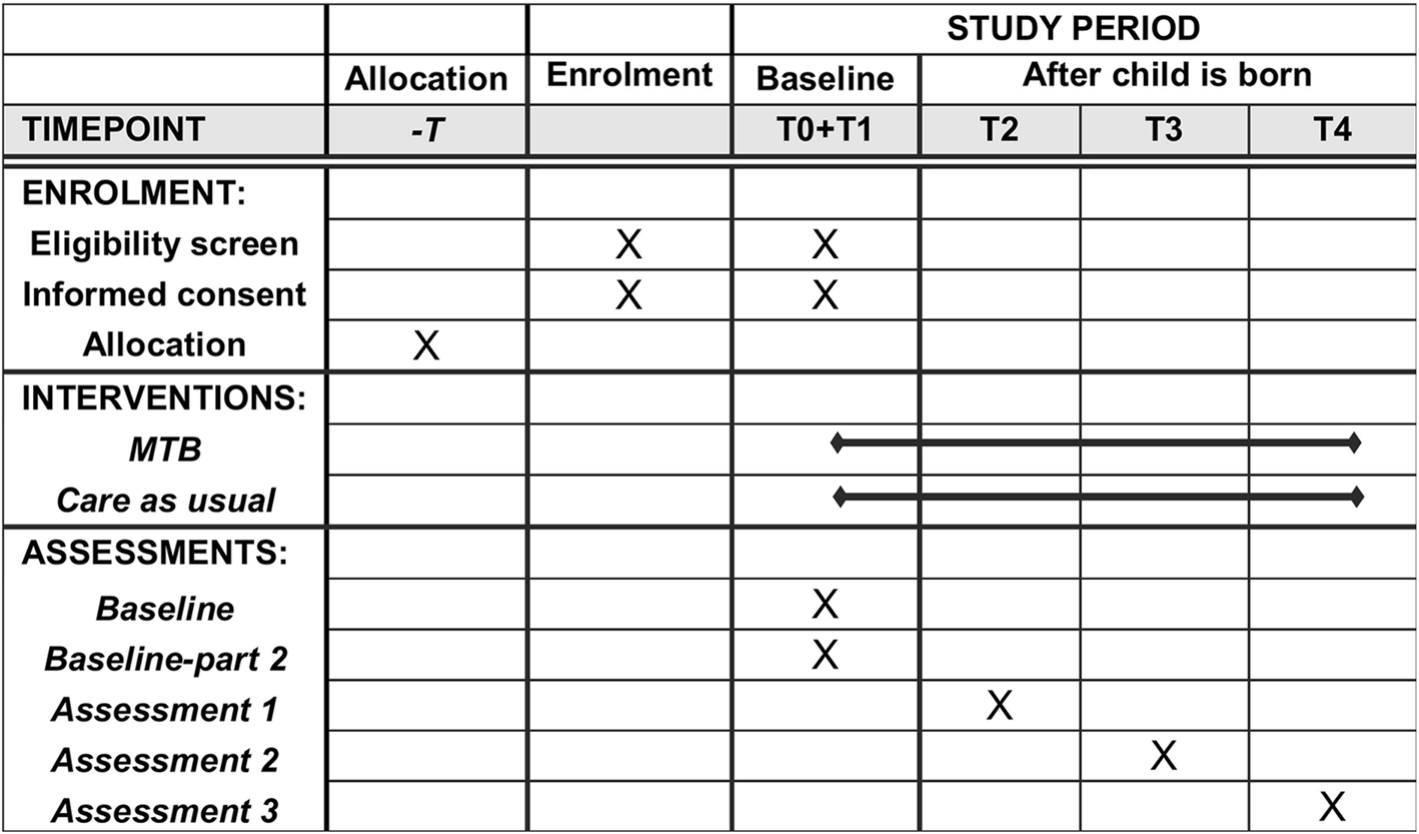
Schedule of enrolment, allocation, intervention, and assessments

The protocol conforms to the Standard Protocol Items: Recommendations for Interventional Trials (SPIRIT) guidelines (Fig. 2).

### Sample size {14}

Power analysis was carried out in the design phase to assess the statistical power for testing the main hypothesis. The power calculation is based on a meta-analysis of interventions aimed at improving parenting sensitivity [57]. The overall average effect size was 0.44 (standardized mean difference, SMD). We expect controls to constitute about one-third of the sample. For normally distributed outcomes and using a two-sided alpha of 0.05, and a beta of 80%, we would need a total of 183 participants (61 control and 122 intervention) for an effect size of 0.44 SMD. To leave room for dropout, we plan to recruit 250 participants (75 control and 175 intervention).

### Recruitment {15}

Participants will be recruited by municipal frontline workers by phone or in person. The municipalities get information about pregnant women in care groups 3 and 4 from the maternity wards, from municipal interdisciplinary meetings about pregnant women at higher risk of adversity, or from child protective services if they receive a reporting about the family or if the pregnant woman previously has been supported by child protective services. Families in the target group who are eligible for the MTB intervention will be invited to participate in the study and will be offered either MTB or the control condition. The frontline worker will give oral information about the study and give the study leaflet to the family. If the pregnant woman gives consent to participate, her partner is also be invited to participate. The consent form and other related documentation given to participants are available from the authors on request.

After receiving consent, the frontline worker will register the pregnant woman (and partner) through a registration form in SurveyXact. Research staff at VIVE then send the baseline questionnaire to the participant (and partner) by text message and by email. Clinicians ask the participant (and partner) to fill out the second part of the baseline questionnaire (childhood trauma experiences and PTSD symptoms) at a home visit and will conduct the Pregnancy Interview with the intervention mothers and partners. Frontline staff will inform VIVE when the child is born.

### Assignment of interventions: allocation

#### Sequence generation {16a}

Participating municipalities were randomized on a site level to take part in either the first wave (2018) or the second wave (2019) of training and implementation of the intervention. Seven municipalities that constitute the original sample were randomized in 2018 to receive MTB training in either 2018 or 2019. Six municipalities constituted a site each, while the seventh, a larger municipality supplied four teams that were randomized as four additional sites. We, therefore, randomized 10 units. The municipalities were informed about allocation immediately after the randomization was conducted.

#### Concealment mechanism {16b}

Each municipality was given a letter (A, B, C, D, E, and F), and the four teams in the larger municipality (G) were numbered from 1 to 4. We applied block randomization with fixed block sizes: the first six municipalities were allocated in a 2:1 ratio so that two municipalities were assigned in the first wave, and four were assigned to the second wave. The uneven allocation ratio was the result of restrictions in training capacity in the initial study phase. For site G with four teams, we received the names of the clinicians in each of the four teams before randomizing the teams 1:1 to either the first or the second wave of training.

#### Implementation {16c}

Randomization was conducted by the trial statistician. Allocations of individual mothers are contingent on the wave a given site is allocated to. All seven original municipalities recruited CAU families before they received training. To secure enough CAU families, most municipalities continued to recruit CAU participants after receiving MTB training. This is the case if the MTB team is full or if the family is recruited very close to term.

Because recruitment was slower than expected and one of the seven original municipalities dropped out before training in 2019, we had to add four municipalities to the study in 2019 and 2020. These four municipalities could not be randomized because there was only one training session available when they joined the study. One municipality that had been trained in MTB before the trial started was added in 2019. This municipality did not have a CAU treatment and therefore only supplied MTB families to the study. One municipality was recruited before training in 2019 (wave 2) and two municipalities were recruited in 2020 where an extra training session was offered due to external funding (wave 3). The three municipalities recruited control families before training, and recruited intervention and control families after receiving training following the same guidelines as the original municipalities.

The municipality that joined before the 2019 training decided to discontinue the MTB intervention. They will continue the MTB intervention until the recruited intervention families graduate and will continue recruiting CAU families for the study.

### Assignment of interventions: blinding

#### Who will be blinded {17a}

As the intervention is a home visiting intervention, participants and care providers cannot be blinded. Outcome assessors, coders, and data analysts will be blinded.

#### Procedure for unblinding if needed {17b}

N/A as participants and clinicians cannot be blinded to treatment allocation.

### Data collection and management

#### Plans for assessment and collection of outcomes {18a}

Data are collected at five time points: T0: baseline immediately after recruitment, T1: baseline part two before the child is born, T2: when the infant is 3 months old, T3: when the infant is 12 months old, and T4: when the infant is 24 months old.

Data are collected through a secured online survey database (SurveyXact). Participants receive a text message or an email with a direct link to the questionnaire. They receive two automatic reminders and additional reminders if needed. The second part of the baseline questionnaire at T1 is collected at a home visit and includes questions about adverse childhood experiences and PTSD symptoms. At T3 and T4, when the child is 12 and 24 months old, mothers are also asked to record and upload a 6-min video of mother and child playing together. The video can be recorded by the family, at a home visit by clinicians from the municipality or trial staff, or through a Zoom-meeting with trial staff.

#### Plans to promote participant retention and complete follow-up {18b}

If the mothers need help to fill out the questionnaire, they can get support from a member of the trial staff or help from a clinician. Mothers receive a DKK 150 (~EUR 20) electronic gift card at each of the four data collections (baseline T0+T1, T2, T3, and T4). They will receive an additional DKK 300 (~EUR 40) for providing a video at T3 and T4. The research team will closely monitor the data collection process and send a monthly status report on data collection to all municipalities. As part of their monitoring of the data collection, the research team contacts participants by text message and phone calls.

The research team works closely with the clinicians in the municipalities to keep participants in the study and to collect data. Families who discontinue the intervention will still be kept in the study unless they specifically express that they wish to withdraw from the study.

#### Data management {19}

Municipal frontline workers enter the participants into a secured online survey database where after research staff enters the participant information into a database kept at a server at the Danish Agency for Governmental IT Services. The data platform conforms to the international ISO27001 standard on how to manage information security. Questionnaire data will be collected directly from the participants through a secured online survey database and will afterwards be transferred to the secure server.

#### Confidentiality {27}

All data are subject to confidentiality and will be handled in accordance with the General Data Protection Regulation (GDPR) and with the Danish legal regulations regarding data protection and security. Access to the data will be strictly limited to members of the research team.

#### Plans for collection, laboratory evaluation, and storage of biological specimens for genetic or molecular analysis in this trial/future use {33}

N/A as we do not collect any biological data.

### Statistical methods

#### Statistical methods for primary and secondary outcomes {20a}

The treatment effect of MTB relative to CAU on the primary outcome (parental sensitivity at child age 12 and 24 months) is analyzed as the mean change from baseline to post-intervention using ordinary least squares regressions separately. The analysis will be adjusted for stratification variables (municipality) and the baseline value of the outcome variable. Variables with indications (*p* < 0.1) of differences between intervention and CAU groups at baseline are also used as control variables. The treatment effect is estimated as a fixed effect using a binary indicator of MTB treatment, and cluster-robust standard errors based on wild bootstrap are used for inference. We evaluate the longitudinal effects separately for the 12-month and 24-month measurements. Two-sided tests with 0.05 significance levels are applied throughout. Analyses of secondary outcomes will be conducted analogously with follow-up analysis at child age 3 months in addition.

Additional exploratory analyses will be carried out in a generalized linear mixed models framework permitting the estimation of flexible nonlinear time patterns to examine the potential heterogeneity of treatment effect sizes across follow-up points.

To avoid the inflation of type 1 error due to multiple outcomes being tested simultaneously, we calculate the false discovery rate (FDR) on the secondary outcomes. In addition, we perform a sensitivity analysis using a Seeming Unrelated Regression framework to collectively examine treatment across all primary and secondary outcomes at age 12-month and 24-month follow-up, hence accounting for the covariance of outcomes.

#### Interim analyses {21b}

There will be no interim analysis.

#### Methods for additional analyses (e.g., subgroup analyses) {20b}

In addition to the primary analysis, we will perform subgroup analyses to examine potential differences between subsets of participants. We will analyze subgroups according to the following characteristics: maternal age (<25 compared to ≥25), primiparous or multiparous, education (less than high school versus high school or more), adult attachment style (ECR-S), single parent or cohabiting, initial trauma level (above cutoff or not on subscales), the initial level of reflective function (lowest 50% versus highest 50%), the initial level of depression or anxiety (clinical or not-clinical level), financial strain (report bad economy or high worry about the economy), and attendance (dose).

We will also examine whether there are differential effects concerning two different composite variables where we combine some of the abovementioned variables: (1) Social determinants of health (based on poverty, low levels of education, single parenthood, and low maternal age); and (2) Low psychological resources (based on depression, anxiety or trauma experience).

For attendance, we will examine if there are differential effects concerning (1) the number of sessions, (2) parents who have participated in the MTB intervention until the child is 1 year old, and (3) light participation (<45 sessions), planned participation (45–90 sessions), and heavy participation (more than 90 sessions).

#### Methods in analysis to handle protocol non-adherence and any statistical methods to handle missing data {20c}

The primary analysis will be based on the intention-to-treat (ITT) principle, aiming to include all participants in the arm they were originally allocated to irrespective of the amount of treatment received. Missing data is handled using multiple imputations using all available baseline data. Sensitivity analyses will be performed to investigate the potential impact of missing data, in particular by using a pre-specified conservative multiple imputation strategy and a complete case analysis.

#### Plans to give access to the full protocol, participant-level data, and statistical code {31c}

To protect participant privacy, the de-identified datasets generated and analyzed during the current study will not be publicly available, but will be available from the corresponding author on reasonable request.

### Oversight and monitoring

#### Composition of the coordinating center and trial steering committee {5d}

VIVE will monitor the study to ensure adherence to ethical aspects, participant rights, and quality of data documentation. The first author (MP) is the primary investigator (PI) and project manager. Day-to-day support is provided by MP, MFH, the trial statistician MT, and a research assistant. The trial does not have a trial steering committee.

#### Composition of the data monitoring committee, its role and reporting structure {21a}

Adverse effects of the intervention are not anticipated; thus, no data monitoring committee is needed in this study.

#### Adverse event reporting and harms {22}

Based on previous studies, we do not anticipate and adverse events. However, if any adverse events occur, they will be reported to the Institutional Review Board at VIVE.

#### Frequency and plans for auditing trial conduct {23}

During the study, the PI meets with each municipality to monitor participant safety and data assessment procedures. The project management group meets monthly to review trial conduct.

#### Plans for communicating important protocol amendments to relevant parties (e.g., trial participants, ethical committees) {25}

If there would be any further necessary protocol amendments a revised copy will be stored and the protocol in the clinical trial registry will be updated. Any amendments or changes will also be transparently described in the publications following the trial.

#### Dissemination plans {31a}

Results will be published in peer-reviewed journals of general and special interest and presented at international conferences. Authorship will follow the Vancouver guidelines. To disseminate the results beyond the scientific community, we will write Danish reports aimed at practitioners and policymakers and present results at the VIVE webpage and through social media platforms.

## Discussion

This paper describes the protocol for a quasi-cluster-randomized controlled trial that aims to examine the effects of the Minding the Baby® home visiting intervention offered to pregnant women at increased risk of adversity on parental sensitivity, parent-child interaction, parental reflective functioning, parental mental health, maternal satisfaction, parental stress, and child development and well-being. This study will provide knowledge on how the MTB intervention works in a Scandinavian context and how to best support families with complex problems in pregnancy and the first years of life. The study will include the largest sample of MTB families to date, increasing the power of the study to detect differences between intervention and control families.

The Scandinavian countries are characterized by universal access to a wide selection of services for all of its citizens (including free and universal health care, birth preparation, generous parental leave, subsidized universal daycare, attractive ratios of children to caregiving staff, free access to schooling and education) [107]. The emphasis on preventive care and existing health and mental health infrastructure in Danish municipalities allow for implementation and potential sustainability of programs such as MTB. This study is the first study implementing MTB in a Scandinavian welfare context and therefore provides important knowledge about the transportability of the MTB intervention. If MTB is shown to be effective, the intervention can be directly implemented into standard care in Danish municipalities.

When conducting studies on families at increased risk for adversity, problems with attendance and attrition are common and inevitable [108]. Socially disadvantaged groups are less likely to engage in interventions and are often described as a “hard to reach” population for intervention studies [109]. However, poor recruitment or retention can reduce the power of a study significantly and lead to inconclusive results and issues around the generalisability of findings if the participants become a non-random subsample of the target population [110–112]. Families at increased risk for adversity may experience challenging life circumstances and complex health and social problems and do not always trust social services and other professionals [113]. Pregnancy can be a window of opportunity for intervening because the pregnant woman usually strongly wishes to be a good mother for her child [114]. However, pregnancy can also complicate the relationship with the professionals because families at increased risk of adversity fear that their child will be removed from their care if they disclose worries or challenges.

To increase attendance and reduce attrition interventions aimed at families at increased risk of adversity must be tailored to the individual family and flexible to accommodate the needs of the study population [108]. To successfully recruit parents at increased risk of adversity, it is important to offer multiple communication channels for recruitment and data collection, to offer well-integrated services and face-to-face contact and it is crucial to plan for attrition and collect data on attendance throughout intervention delivery [108].

As the target population for MTB is at increased risk for adversity, it is not surprising that the previous studies have faced challenges with recruitment and retention. Attrition rates in the two studies by the developers of the intervention ranged from 20 to 29% at 12- and 24-month assessments [61, 63, 64]. The recent study in the UK struggled with recruitment and had to downscale the study from 200 to 150 participants due to recruitment challenges [67]. The attrition rates for the present study are 27% at 6 months, 34% at 12 months, and 35% at the 2-year assessment. These problems with recruitment and retention may make it impossible to detect intervention effects smaller than *d* = .40–.50, which would include effect sizes of potential clinical significance.

In this study, the process for recruitment and data collection has been tailored to this exact group of pregnant women to increase the chance of recruiting the planned number of participants and retaining them in the study. Currently, we have collected baseline measures for 254 participants making this study the largest study of MTB. Maternal sensitivity is the primary outcome of the study and collecting video recordings from the participants is therefore pivotal. Some women refuse to submit a video recording at child age 12 and 24 months, perhaps because they fear being judged as a parent. To collect as many videos as possible, we have developed different possible procedures for video recordings (including home visits by either a known person or by independent trial staff), recording the video by the participant without any help or recording it by zoom with trial staff) and have produced a short informational video about the video recording. We will closely monitor referral, recruitment, and retention rates through the study and accommodate procedures if needed.

The study has recruited participants from June 2018 and will continue to recruit until the end of May 2022. This is a period where several historical events have taken place and impacted the study. GDPR was introduced in Europe in May 2018 right before recruitment started with many new laws concerning processing personal data for research purposes making it difficult to produce recruitment materials. Consequently, it took longer time to finalize data agreements with several municipalities. The focus on data leaks and the importance of the GDPR also made some participants wary of handing over sensitive and personal information. The Covid-19 pandemic also has affected the trial. Since March 2019, Denmark has experienced different levels of lockdown and restrictions challenging recruitment, intervention, and outcome assessment. Furthermore, Denmark experienced a strike among nurses from June 19 to August 28, 2021, where some of the health nurses were affected.

In sum, there is a need for early interdisciplinary interventions for parents and infants at increased risk for adversity. The present study of MTB in a Scandinavian context has the potential to improve the well-being of parents and infants at increased risk for adversity and to prevent health inequalities in the long run.

### Trial status

This protocol is version 1.4. Recruitment started on May 28th, 2018, and is expected to be completed in May 2022.

## Data Availability

Only the research staff at VIVE (including MP, MFH, and MT) will have access to the final dataset. The datasets generated and analyzed during the current study are not publicly available to protect participant privacy but are available from the corresponding author on reasonable request.

## Abbreviations

MTB: Minding the Baby®
CIB: Coding Interactive Behavior
RCT: Randomized controlled trial
CAU: Care as usual
COS-P: Circle of Security – Parenting
P-PRFQ: Prenatal Parental reflective functioning questionnaire
HADS: Hospital Anxiety and Depression Scale
ECR-S: Experiences in Close Relationship Scale-Short Form
CTQ: Childhood trauma questionnaire
WEMWBS: Warwick-Edinburgh Mental Well-being Scale
WHO-5: The World Health Organization (WHO)-5 Well-Being Index
EPDS: Edinburgh Postnatal Depression Scale
BAM-13: Being a Mother
PRFQ: Parental reflective functioning questionnaire
PSS: The Parenting Stress Scale
CSI: Couple Satisfaction Index
ASQ:3: Ages and Stages Questionnaire 3
ASQ:SE-2: Ages and Stages Questionnaire-Social Emotional 2
SDQ: The Strengths and Difficulties Questionnaire
MABISC: The Mother and Baby Interaction Scale
MIRS: The Mother-Infant Relationship Scale
DREAM: Danish Rational Economic Agents Model
SMD: Standardized mean difference
PTSD: Posttraumatic stress syndrome disorder
GDPR: General Data Protection Regulation
FDR: False discovery rate

## References

[1] Heckman JJ. The case for investing in young children. Big Ideas Child Invest Our Nation’s Fut. 2012:49–58 Available from: https://firstfocus.org/wp-content/uploads/2014/06/Big-Ideas-2008.pdf.

[2] Shonkoff JP, Slopen N, Williams DR (2020). Early childhood adversity, toxic stress, and the impacts of racism on the foundations of health. Annu Rev Public Health.

[3] Rod NH, Bengtsson J, Elsenburg LK, Taylor-Robinson D, Rieckmann A (2021). Hospitalisation patterns among children exposed to childhood adversity: a population-based cohort study of half a million children. Lancet Public Health.

[4] Davidson RJ, Mcewen BS (2012). Social influences on neuroplasticity : stress and interventions to promote well-being. Nat Neurosci.

[5] Shonkoff JP (2011). Protecting brains, not simply stimulating minds. Science (80- ).

[6] Murray L (2014). The Psychology of Babies: how relationships support development from birth to two.

[7] Kefeli MC, Turow RG, Yıldırım A, Boysan M (2018). Childhood maltreatment is associated with attachment insecurities, dissociation and alexithymia in bipolar disorder. Psychiatry Res.

[8] Hughes K, Bellis MA, Hardcastle KA, Sethi D, Butchart A, Mikton C (2017). The effect of multiple adverse childhood experiences on health: a systematic review and meta-analysis. Lancet Public Health.

[9] Holt S, Buckley H, Whelan S (2008). The impact of exposure to domestic violence on children and young people: A review of the literature. Child Abuse Negl.

[10] Doyle O (2020). The first 2,000 days and child skills. J Polit Econ.

[11] Almond D, Currie J, Duque V (2018). Childhood circumstances and adult outcomes: Act II. J Econ Lit.

[12] Boyce WT, Levitt P, Martinez FD, McEwen BS, Shonkoff JP (2021). Genes, environments, and time: the biology of adversity and resilience. Pediatrics..

[13] National Scientific Council on the Developing Child. Excessive stress disrupts the architecture of the developing brain: Working Paper 3. Work Pap. 2014:1–12.

[14] Cassidy J, Cassidy J, Shaver PR (2018). The nature of the child’s ties. Handb Attach theory, Res Clin Appl Res Clin Appl. Third.

[15] De Wolff MS, van Ijzendoorn MH (1997). Sensitivity and attachment: a meta-analysis on parental antecedents of infant attachment. Child Dev.

[16] Feldman R (2015). Mutual influences between child emotion regulation and parent–child reciprocity support development across the first 10 years of life: Implications for developmental psychopathology. Dev Psychopathol.

[17] Bakermans-Kranenburg MJ, van IJzendoorn MH. (2009). The first 10,000 Adult Attachment Interviews: distributions of adult attachment representations in clinical and non-clinical groups. Attach Hum Dev.

[18] Gentile S (2017). Untreated depression during pregnancy: short- and long-term effects in offspring. A systematic review. Neuroscience.

[19] Netsi E, Pearson RM, Murray L, Cooper P, Craske MG, Stein A (2018). Association of persistent and severe postnatal depression with child outcomes. JAMA Psychiat.

[20] Rayce SB, Rasmussen IS, Væver MS, Pontoppidan M (2020). Effects of parenting interventions for mothers with depressive symptoms and an infant: systematic review and meta-analysis. BJPsych Open.

[21] van Doesum KTM, Riksen-Walraven JM, Hosman CMH, Hoefnagels C (2008). A randomized controlled trial of a home-visiting intervention aimed at preventing relationship problems in depressed mothers and their infants. Child Dev.

[22] Lawler JM, Bocknek EL, McGinnis EW, Martinez-Torteya C, Rosenblum KL, Muzik M (2019). Maternal postpartum depression increases vulnerability for toddler behavior problems through infant cortisol reactivity. Infancy..

[23] Williams RC, Biscaro A, Clinton J (2019). Relationships matter: How clinicians can support positive parenting in the early years. Paediatr Child Health.

[24] Bowlby J (1958). The nature of the child’s tie to his mother. Int J Psychoanal.

[25] Ainsworth MS (1979). Infant-mother attachment. Am Psychol.

[26] Belsky J, Fearon RMP, Cassidy J, Shaver PR (2018). Precursors of attachment security. Handb Attach theory, Res Clin Appl.

[27] Ainsworth MDS, Bell SM, Stayton DF. Infant-mother attachment and social development: Socialization as a product of reciprocal responsiveness to signals: Cambridge University Press; 1974.

[28] Deans CL (2020). Maternal sensitivity, its relationship with child outcomes, and interventions that address it: a systematic literature review. Early Child Dev Care.

[29] Hoeve M, Dubas JS, Eichelsheim VI, van der Laan PH, Smeenk W, Gerris JRM (2009). The relationship between parenting and delinquency: a meta-analysis. J Abnorm Child Psychol.

[30] Majumder MA (2016). The impact of parenting style on children’s educational outcomes in the United States. J Fam Econ Issues.

[31] Puckering C, Allely CS, Doolin O, Purves D, McConnachie A, Johnson PCD (2014). Association between parent-infant interactions in infancy and disruptive behaviour disorders at age seven: a nested, case–control ALSPAC study. BMC Pediatr.

[32] Wilson P, Bradshaw P, Tipping S, Henderson M, Der G, Minnis H (2013). What predicts persistent early conduct problems? Evidence from the Growing Up in Scotland cohort. J Epidemiol Community Health.

[33] Fearon RP, Bakermans-Kranenburg MJ, van Ijzendoorn MH, Lapsley A-M, Roisman GI (2010). The significance of insecure attachment and disorganization in the development of children’s externalizing behavior: a meta-analytic study. Child Dev.

[34] Madigan S, Atkinson L, Laurin K, Benoit D (2013). Attachment and internalizing behavior in early childhood: A meta-analysis. Dev Psychol.

[35] Allely CS, Purves D, McConnachie A, Marwick H, Johnson P, Doolin O (2013). Parent-infant vocalisations at 12 months predict psychopathology at 7 years. Res Dev Disabil.

[36] Barlow J, Coren E (2018). The effectiveness of parenting programs. Res Soc Work Pract.

[37] Doyle O, Harmon C, Heckman JJ, Logue C, Hyeok S (2017). Early skill formation and the e ffi ciency of parental investment : a randomized controlled trial of home visiting. Labour Econ.

[38] Leijten P, Gardner F, Landau S, Harris V, Mann J, Hutchings J, et al. Research review: harnessing the power of individual participant data in a meta-analysis of the benefits and harms of the Incredible Years parenting program. J Child Psychol Psychiatry. 2017; Available from: http://doi.wiley.com/10.1111/jcpp.12781. 10.1111/jcpp.12781 28696032

[39] Rayce SB, Rasmussen IS, Klest SK, Patras J, Pontoppidan M (2017). Effects of parenting interventions for at-risk parents with infants: a systematic review and meta-analyses. BMJ Open.

[40] Chen M, Chan KL (2016). Effects of parenting programs on child maltreatment prevention. Trauma Violence Abuse.

[41] Olds DL, Sadler L, Kitzman H (2007). Programs for parents of infants and toddlers: recent evidence from randomized trials. J Child Psychol Psychiatry.

[42] Furlong M, McGilloway S, Bywater T, Hutchings J, Smith SM, Donnelly M (2013). Behavioural and cognitive-behavioural group-based parenting programmes for early-onset conduct problems in children aged 3 to 12 years’. Evid-Based Child Heal.

[43] Welsh BC, Farrington DP (2007). Scientific support for early prevention of delinquency and later offending. Vict Offenders.

[44] Heckman JJ, Masterov DV (2007). The productivity argument for investing in young children. Rev Agric Econ.

[45] Reedtz C, Handegård BH, Mørch W-T (2011). Promoting positive parenting practices in primary pare: outcomes and mechanisms of change in a randomized controlled risk reduction trial. Scand J Psychol.

[46] Piquero AR, Jennings WG, Diamond B, Farrington DP, Tremblay RE, Welsh BC (2016). A meta-analysis update on the effects of early family/parent training programs on antisocial behavior and delinquency. J Exp Criminol.

[47] Heckman JJ, Darling-Hammond L, Grunewald R, Heckman JJ, Isaacs JB, Kirp DL, Rolnick AJ (2008). The case for investing in disadvantaged young children. Big Ideas Child Invest Our Nation’s Futur.

[48] Bachmann CJ, Beecham J, O’Connor TG, Briskman J, Scott S. A good investment: longer-term cost savings of sensitive parenting in childhood. J Child Psychol Psychiatry Allied Discip. 2021. 10.1111/jcpp.13461 34187093

[49] Barlow J, Bergman H, Kornør H, Wei Y, Bennett C. Group-based parent training programmes for improving emotional and behavioural adjustment in young children. Cochrane Database Syst Rev. 2016; Available from: https://doi.wiley.com/10.1002/14651858.CD003680.pub3. 10.1002/14651858.CD003680.pub3 PMC6797064 27478983

[50] Feldman R, Eidelman AI (2006). Neonatal state organization, neuromaturation, mother-infant interaction, and cognitive development in small-for-gestational-age premature infants. Pediatrics..

[51] Feldman R, Weller A, Sirota L, Eidelman AI (2003). Testing a family intervention hypothesis: the contribution of mother-infant skin-to-skin contact (kangaroo care) to family interaction, proximity, and touch. J Fam Psychol.

[52] Kristensen IH, Simonsen M, Trillingsgaard T, Kronborg H (2017). Video feedback promotes relations between infants and vulnerable first-time mothers: a quasi-experimental study. BMC Pregnancy Childbirth.

[53] MacBeth A, Law J, McGowan I, Norrie J, Thompson L, Wilson P (2015). Mellow Parenting: systematic review and meta-analysis of an intervention to promote sensitive parenting. Dev Med Child Neurol.

[54] Mountain G, Cahill J, Thorpe H (2017). Sensitivity and attachment interventions in early childhood: a systematic review and meta-analysis. Infant Behav Dev.

[55] Granqvist P, Sroufe LA, Dozier M, Hesse E, Steele M, van Ijzendoorn M (2017). Disorganized attachment in infancy: a review of the phenomenon and its implications for clinicians and policy-makers. Attach Hum Dev.

[56] Zajac L, Raby KL, Dozier M. Sustained effects on attachment security in middle childhood: results from a randomized clinical trial of the Attachment and Biobehavioral Catch-up (ABC) intervention. J Child Psychol Psychiatry. 2020;61:417–24 Available from: https://onlinelibrary.wiley.com/doi/10.1111/jcpp.13146. 10.1111/jcpp.13146 PMC7135967 31677152

[57] Bakermans-Kranenburg MJ, van IJzendoorn MH, Juffer F. Less is more: meta-analyses of sensitivity and attachment interventions in early childhood. Psychol Bull. 2003;129:–195, 215 Available from: http://doi.apa.org/getdoi.cfm?doi=10.1037/0033-2909.129.2.195. 10.1037/0033-2909.129.2.195 12696839

[58] Slade A (2005). Parental reflective functioning: an introduction. Attach Hum Dev.

[59] Slade A, Grienenberger J, Bernbach E, Levy D, Locker A (2005). Maternal reflective functioning, attachment, and the transmission gap: a preliminary study. Attach Hum Dev.

[60] Kelly K, Slade A, Grienenberger JF (2005). Maternal reflective functioning, mother–infant affective communication, and infant attachment: Exploring the link between mental states and observed caregiving behavior in the intergenerational transmission of attachment. Attach Hum Dev.

[61] Slade A, Holland ML, Ordway MR, Carlson EA, Jeon S, Close N (2020). Minding the Baby ®: enhancing parental reflective functioning and infant attachment in an attachment-based, interdisciplinary home visiting program. Dev Psychopathol.

[62] Allen JG, Fonagy P. Handbook of mentalization-based treatment. Handb Ment Treat. 2008:1–340.

[63] Sadler LS, Slade A, Close N, Webb DL, Simpson T, Fennie K (2013). Minding the Baby: enhancing reflectiveness to improve early health and relationship outcomes in an interdisciplinary home-visiting program. Infant Ment Health J.

[64] Ordway MR, Sadler LS, Dixon J, Close N, Mayes L, Slade A (2014). Lasting effects of an interdisciplinary home visiting program on child behavior: preliminary follow-up results of a randomized trial. J Pediatr Nurs.

[65] Ordway MR, Sadler LS, Holland ML, Slade A, Close N, Mayes LC. A home visiting parenting program and child obesity: a randomized trial. Pediatrics. 2018;141 Available from: https://publications.aap.org/pediatrics/article/141/2/e20171076/38018/A-Home-Visiting-Parenting-Program-and-Child. 10.1542/peds.2017-1076 PMC5810599 29339565

[66] Londono Tobon A, Condon E, Sadler LS, Holland ML, Mayes LC, Slade A (2022). School age effects of Minding the Baby—an attachment-based home-visiting intervention—On parenting and child behaviors. Dev Psychopathol.

[67] Longhi E, Murray L, Wellsted D, Hunter R, Mackenzie K, Taylor-Colls S, et al. Minding the Baby® Home-visiting programme for vulnerable young mothers: results of a randomised controlled trial in the UK: National Society for the Prevention of Cruelty to Children; 2019.

[68] Denmark S (2020). Statistikbanken.

[69] Sundhedsstyrelsen. Anbefalinger for svangreomsorgen. 2013.

[70] Aabakke AJM, Mortensen LH, Krebs LH (2019). Socioøkonomiske faktorer har betydning for graviditet og fødsel. Ugeskr Laeger.

[71] Wendell AD (2013). Overview and epidemiology of substance abuse in pregnancy. Clin Obstet Gynecol.

[72] Zhong QY, Gelaye B, Fricchione GL, Avillach P, Karlson EW, Williams MA (2018). Adverse obstetric and neonatal outcomes complicated by psychosis among pregnant women in the United States. BMC Pregnancy Childbirth. BMC Pregnancy Childbirth.

[73] Sundhedsstyrelsen (2021). Anbefalinger for svangreomsorgen. Sundhedsstyrelsen.

[74] Sundhedsstyrelsen (2019). Vejledning om forebyggende sundhedsydelser til børn og unge.

[75] Slade A, Simpson TE, Webb D, Albertson JG, Close N, Sadler L, Steele H, Steele M (2017). Minding the Baby: complex trauma and attachment-based home intervention. Handb Attach Interv.

[76] Pajulo M, Tolvanen M, Karlsson L, Halme-Chowdhury E, Öst C, Luyten P (2015). The prenatal parental reflective functioning questionnaire: exploring factor structure and construct validity of a new measure in the finn brain birth cohort pilot study. Infant Ment Health J.

[77] Luyten P, Mayes LC, Nijssens L, Fonagy P (2017). The parental reflective functioning questionnaire: Development and preliminary validation. Eapen V, editor. PLoS One.

[78] Bjelland I, Dahl AA, Haug TT, Neckelmann D (2002). The validity of the Hospital Anxiety and Depression Scale. J Psychosom Res.

[79] Zigmond AS, Snaith RP (1983). The hospital anxiety and depression scale. Acta Psychiatr Scand.

[80] Wei M, Russell DW, Mallinckrodt B, Vogel DL (2007). The Experiences in Close Relationship Scale (ECR)-Short Form: reliability, validity, and factor structure. J Pers Assess.

[81] James AC, James G, Cowdrey FA, Soler A, Choke A, Ac J (2015). Cognitive behavioural therapy for anxiety disorders in children and adolescents ( Review ). Cochrane Database Syst Rev.

[82] Hansen M, Andersen TE, Armour C, Elklit A, Palic S, Mackrill T (2010). PTSD-8: A Short PTSD Inventory. Clin Pract Epidemiol Ment Health.

[83] Feldman R. Parenting behavior as the environment where children grow. In: Mayes L, Lewis M, editors. Cambridge Handb Environ Hum Dev: Cambridge University Press; 2012. p. 535–67.

[84] Feldman R. The relational basis of adolescent adjustment: trajectories of mother–child interactive behaviors from infancy to adolescence shape adolescents’ adaptation. Attach Hum Dev. 2010;12:173–92 Available from: http://www.tandfonline.com/doi/abs/10.1080/14616730903282472. 10.1080/14616730903282472 20390528

[85] Feldman R, Bamberger E, Kanat-Maymon Y (2013). Parent-specific reciprocity from infancy to adolescence shapes children’s social competence and dialogical skills. Attach Hum Dev.

[86] Koushede V, Lasgaard M, Hinrichsen C, Meilstrup C, Nielsen L, Rayce SB (2019). Measuring mental well-being in Denmark: validation of the original and short version of the Warwick-Edinburgh mental well-being scale (WEMWBS and SWEMWBS) and cross-cultural comparison across four European settings. Psychiatry Res.

[87] Stewart-Brown S, Tennant A, Tennant R, Platt S, Parkinson J, Weich S (2009). Internal construct validity of the Warwick-Edinburgh Mental Well-Being Scale (WEMWBS): a Rasch analysis using data from the Scottish Health Education Population Survey. Health Qual Life Outcomes.

[88] Bech P. Measuring the Dimension of Psychological General Well-Being by the WHO-5. Qual Life Newsl. 2004:16–6.

[89] Bech P (2011). Klinisk psykometri.

[90] Smith-Nielsen J, Matthey S, Lange T, Væver MS (2018). Validation of the Edinburgh Postnatal Depression Scale against both DSM-5 and ICD-10 diagnostic criteria for depression. BMC Psychiatry. BMC Psychiatry.

[91] Cox JL, Holden JM, Sagovsky R (1987). Edinburgh Postnatal Depression Scale 1 (EPDS) Instructions for using the Edinburgh Postnatal Depression Scale. Br J Psychiatry.

[92] Matthey S (2011). Assessing the experience of motherhood: The Being a Mother Scale (BaM-13). J Affect Disord.

[93] Berry JO, Jones WH (1995). The Parental Stress Scale: initial psychometric evidence. J Soc Pers Relat.

[94] Pontoppidan M, Nielsen T, Kristensen IH (2018). Psychometric properties of the Danish Parental Stress Scale: Rasch analysis in a sample of mothers with infants. Gnambs T, editor. PLoS One.

[95] Funk JL, Rogge RD (2007). Testing the ruler with item response theory: increasing precision of measurement for relationship satisfaction with the Couples Satisfaction Index. J Fam Psychol.

[96] Squires J, Bricker D, Waddell M, Funk K, Clifford J, Hoselton R (2017). Social-Emotional Assessment/Evaluation Measure.

[97] Squires J, Twombly E, Bricker D, Potter L. ASQ-3 User’s Guide: Brookes Publishing; 2009.

[98] Squires J, Twombly E, Bricker D, Potter L. Appendix C - ASQ-3 Technical Report. In: Squires J, Twombly E, Bricker D, Potter L, editors. ASQ®-3 User’s Guid: Brookes Publishing; 2009. p. 147–75.

[99] Squires JK, Bricker DD, Twombly E. Ages & Stages Questionnaires®: Social-Emotional, Second Edition (ASQ®:SE-2): A Parent-Completed Child Monitoring System for Social-Emotional Behaviors: Brookes Publishing; 2015.

[100] Squires J, Bricker D, Twombly E, Murphy K, Hoselton R, Chen Y. Appendix C - ASQ:SE-2 Technical Report. In: Squires J, Bricker D, Twombly E, editors. ASQSE-2^TM^ User’s Guid: Brookes Publishing; 2015. p. 181–208.

[101] Goodman R (2001). Psychometric Properties of the Strengths and Difficulties Questionnaire. J Am Acad Child Adolesc Psychiatry.

[102] Arnfred J, Svendsen K, Rask C, Jeppesen P, Fensbo L, Houmann T, et al. Danish norms for the Strengths and Difficulties Questionnaire. Dan Med J. 2019;66 Available from: http://www.ncbi.nlm.nih.gov/pubmed/31256773. 31256773

[103] Kadesjö B, Janols L-O, Korkman M, Mickelsson K, Strand G, Trillingsgaard A (2018). Manual 5-15R (Five-To-Fifteen-Revised).

[104] Høivik MS, Burkeland NA, Linaker OM, Berg-Nielsen TS (2013). The Mother and Baby Interaction Scale: a valid broadband instrument for efficient screening of postpartum interaction? A preliminary validation in a Norwegian community sample. Scand J Caring Sci.

[105] Newman-Morris V, Gray KM, Simpson K, Newman LK (2020). Development and initial reliability and validity of a new measure of distorted maternal representations: The Mother–Infant Relationship Scale. Infant Ment Health J.

[106] Rosholm M, Sørensen K, Skipper L (2017). BIP Indikatorer og jobsandsynlighed. Hovedpointer.

[107] Rosholm M, Paul A, Bleses D, Højen A, Dale S, Jensen P (2021). Are impacts of early interventions in the Scandinavian Welfare State consistent with a Heckman Curve? A Meta-Analysis. J Econ Surv.

[108] Pote I, Doubell L, Brims L, Larbie J, Stock L, Lewing B, et al. Engaging disadvantaged and vulnerable parents: an evidence review. Early Interv Found. 2019; Available from: https://www.eif.org.uk/files/pdf/engaging-disadvantaged-vulnerable-parents.pdf.

[109] Bonevski B, Randell M, Paul C, Chapman K, Twyman L, Bryant J (2014). Reaching the hard-to-reach: a systematic review of strategies for improving health and medical research with socially disadvantaged groups. BMC Med Res Methodol.

[110] Treweek S, Lockhart P, Pitkethly M, Cook JA, Kjeldstrøm M, Johansen M (2013). Methods to improve recruitment to randomised controlled trials: Cochrane systematic review and meta-analysis. BMJ Open.

[111] Treweek S, Mitchell E, Pitkethly M, Cook J, Kjeldstrøm M, Taskila T, Treweek S (2010). Strategies to improve recruitment to randomised controlled trials. Cochrane Database Syst Rev.

[112] Britton A, McKee M, Black N, McPherson K, Sanderson C, Bain C (1999). Threats to applicability of randomised trials: exclusions and selective participation. J Health Serv Res Policy.

[113] Taplin S. Prenatal reporting to child protection: Characteristics and service responses in one Australian jurisdiction. Child Abuse Negl, Elsevier Ltd. 2017;65:68–76 Available from: 10.1016/j.chiabu.2017.01.007. 10.1016/j.chiabu.2017.01.007 28113086

[114] Olander EK, Darwin ZJ, Atkinson L, Smith DM, Gardner B (2016). Beyond the “teachable moment” - a conceptual analysis of women’s perinatal behaviour change. Women Birth.

